# The Knowledge-to-Practice Gap in Safe Lifting and Biomechanical Risks as Determinants of Musculoskeletal Disorders in Manual Material Handling

**DOI:** 10.3390/ijerph23081041

**Published:** 2026-08-11

**Authors:** Chedsada Saisombut, Pheerasak Assavanopakun, Pevra Kanta, Jinjuta Panumasvivat

**Affiliations:** 1 Department of Community Medicine, Faculty of Medicine, Chiang Mai University, Chiang Mai 50200, Thailand; chedsada.s@cmu.ac.th (C.S.); pheerasak.assava@cmu.ac.th (P.A.); 2 Environmental and Occupational Medicine Excellence Center, Faculty of Medicine, Chiang Mai University, Chiang Mai 50200, Thailand; pevra.k@cmu.ac.th

**Keywords:** work-related musculoskeletal disorders, low back pain, biomechanical risk factors, manual lifting, knowledge attitude and practice

## Abstract

**Highlights:**

**Public health relevance—How does this work relate to a public health issue?**

- MSDs remain a major global disability burden and the most common occupational hazard worldwide.
- Investigating the interplay between workplace risks and individual KAP deepens the occupational health approach to disease prevention.

**Public health significance—Why is this work of significance to public health?**

- Safety knowledge protects against chronic MSDs but fails to mitigate age-related acute risks.
- A critical safe lifting knowledge-to-practice gap leaves manual lifters highly vulnerable to workplace MSDs.

**Public health implications—What are the key implications or messages for practitioners, policy makers and/or researchers in public health?**

- Public health and safety controls must integrate workplace ergonomics, age-tailored controls, and practice-based training.

**Abstract:**

This study investigated the complex interfaces between knowledge, attitude and practice (KAP); biomechanical risks; and musculoskeletal disorders (MSDs) among heavy manual handling workers. A cross-sectional study surveyed 99 industrial manual lifting workers within manufacturing factories (mean age: 33.4 ± 7.7 years; mean job experience: 7.95 ± 6.5 years) using a validated online questionnaire assessing demographics, occupational conditions, biomechanical risk, lifting KAP, and MSD prevalence via the Extended Nordic Musculoskeletal Questionnaire. Associations with 12-month and active 7-day MSD prevalence were analyzed using multinomial logistic regression. The 12-month prevalence of MSDs was 56.6%, predominantly affecting the lower back (51.0%). The MSD group reported significantly higher biomechanical risks. Chi-square analysis revealed significant differences in safety practices (*p* = 0.025), particularly regarding pre-work stretching, load-weight assessment, and overhead lifting. Higher biomechanical risk was significantly associated with 12-month MSDs without 7-day pain (RRR = 1.54, 95% CI: 1.10 to 2.16, *p* = 0.012). Conversely, greater knowledge demonstrated a significant protective association against 12-month MSDs without 7-day pain (RRR = 0.74, 95% CI: 0.56 to 0.99, *p* = 0.045). Age-related vulnerability significantly increased active 7-day MSDs. MSD prevalence is driven by cumulative biomechanical hazards. Although knowledge is associated with lower long-term risk, a critical knowledge-to-practice gap exists. Interventions must prioritize workplace ergonomic risk reduction with age-tailored adjustments. These should be integrated with active, practice-based training to foster behavioral modification.

## 1. Introduction

Work-related musculoskeletal disorders (WMSDs) represent a preeminent global public health challenge, imposing a substantial disease burden [1]. Specifically, low back pain (LBP) has been consistently identified as the leading cause of Years Lived with Disability (YLDs) among the working-age population worldwide. According to the Global Burden of Disease 2021 study, LBP affected approximately 628.8 million individuals and accounted for over 70.1 million YLDs globally [2,3]. Within the Thai context, statistics from the Social Security Office’s Workmen’s Compensation Fund (2020–2024) affirm that WMSDs constitute the most prevalent work-related illness. These disorders not only significantly diminish the quality of life of workers but also precipitate profound health economic losses and reductions in organizational productivity [4].

Industrial workers engaged in heavy manual handling are at a markedly elevated risk of developing WMSDs, particularly LBP and intervertebral disc disorders. Recent systematic reviews and meta-analyses demonstrate robust evidence that cumulative mechanical exposures, predominantly heavy lifting, repetitive tasks, and awkward or non-neutral postures, are significantly associated with the onset of these chronic spinal conditions [5,6]. Although numerous enterprises have implemented occupational health and safety measures, including weightlifting limits, the provision of mechanical aids, and ergonomic workplace modifications, the reliance on manual labor for load handling remains an unavoidable necessity in many actual production processes [7,8]. Furthermore, many workplaces have promoted the use of back support belts among manual lifting workers; however, the current literature lacks clear, conclusive evidence to support their efficacy as genuine protective devices [9].

Biomechanically, safe lifting necessitates the coordinated engagement of core musculature, particularly the lower back, abdominal, and hip muscles [10,11]. Improper postures, such as lumbar flexion without knee bending or torso twisting during load bearing, substantially exacerbate compressive and shear forces on the spine, precipitating tissue injury [10].

Empirical evidence indicates that individuals with chronic LBP frequently exhibit compromised muscle performance, characterized most notably by a significant reduction in static back extensor endurance compared to asymptomatic individuals [11]. Consequently, evaluating functional muscle performance in conjunction with clinical symptoms serves as a critical indicator for identifying physical risks among workers.

Beyond environmental determinants, workers’ knowledge, attitude, and practice (KAP) regarding occupational safety serve as pivotal behavioral variables that dictate the risk of WMSDs. Despite a forementioned lack of evidence regarding back support belts, misconceptions about their utility remain prevalent in occupational settings. Reliance on such passive supports may paradoxically engender a false sense of security, prompting workers to exceed their physical lifting capacities while neglecting proper biomechanics [9]. Current evidence posits that education, or passive support alone, is insufficient for LBP prevention; rather, they must be integrated with behavioral modifications and active exercise programs designed to enhance muscle endurance [7,8]. Specifically, incorporating stretching protocols serves as a critical preventive strategy, as these practices significantly enhance joint range of motion, improve muscle-tendon elasticity, and effectively prevent the onset of WMSDs [6].

Given these knowledge gaps, a comprehensive understanding of KAP regarding manual lifting and back belt use, alongside their interrelationships with physical performance and clinical symptoms, is imperative for designing sustainable, practical interventions. Insights derived from this study will facilitate the development of targeted behavioral risk assessments and promote workplace safety. Therefore, this cross-sectional study primarily aimed to investigate the relationships with KAP regarding manual lifting and back support belt use and the occurrence of musculoskeletal disorders (MSDs) among heavy manual handling workers.

## 2. Materials and Methods

### 2.1. Study Design and Participants

A cross-sectional study was conducted among industrial workers from manufacturing factories in Chiang Mai and Lamphun provinces, whose primary duties consistently involved physically demanding tasks—such as heavy manual lifting, loading, and transferring materials—for at least one year. The study period ranged from 22 July 2025 to 17 March 2026. Inclusion criteria were individuals aged 20 years old or older, working in Chiang Mai or Lamphun provinces, understanding in Thai, and being willing to join the study. Participants with incomplete data were excluded. Sample size was estimated using a one-sample proportion test [12]. The target prevalence (P_1_ = 65.6%) was derived from a recent global meta-analysis of manufacturing workers [13] against a null benchmark proportion (P_0_ = 50%) [14], with a two-sided α = 0.05 and 80% power; the minimum sample size was 79 participants. To account for a potential 25% dropout or incomplete response rate, the target sample size was established at a minimum of 99 participants.

A convenience sampling strategy was employed via open invitations distributed by the occupational health and safety units and human resources departments within each workplace and factory, inviting them to access and complete the online questionnaire platform. A total of 102 workers submitted questionnaires; following the exclusion of 3 incomplete submissions, a final sample of 99 valid responses (*N* = 99) was analyzed. A formal response rate could not be calculated due to open-invitation recruitment.

**Figure 1:**
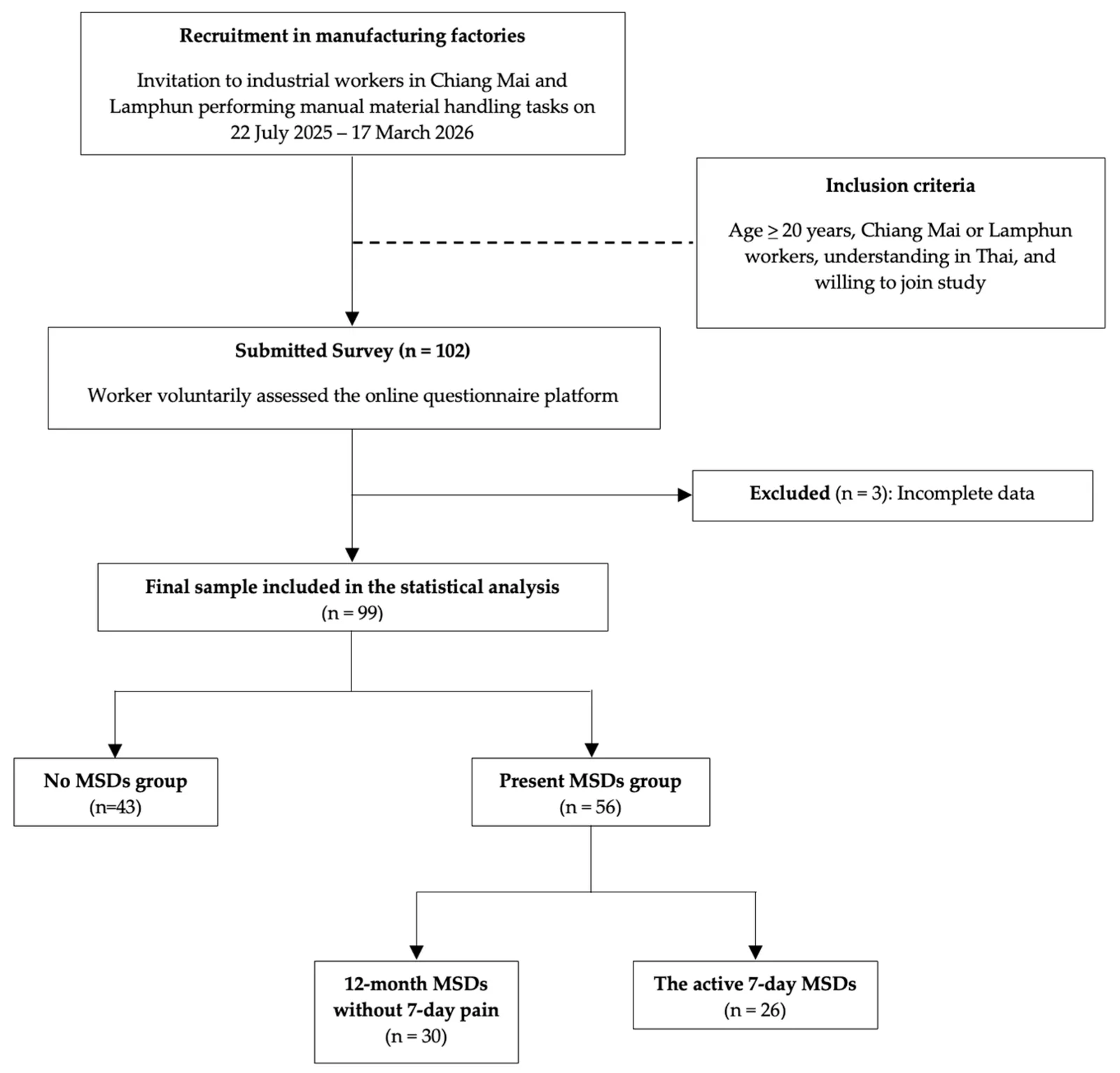
Flowchart of the study process, participant selection, and group categorization.

Figure 1 illustrates the participant recruitment, screening, and final selection process. This study was conducted and reported in accordance with the STROBE guidelines for cross-sectional studies (Supplementary Material File S1).

### 2.2. Data Collection

The self-reported anonymous questionnaire was developed and divided into four sections:

1. Demographic Data: Including age, sex, weight, height, BMI, education level, physical activity, underlying diseases, smoking, and hobbies.
2. Working Conditions: Including working duration, working hours, overtime, secondary job, support equipment, and biomechanical risks in job tasks. Biomechanical risk factors within job tasks were evaluated across nine domains adapted from workplace biomechanics frameworks and previous studies and recommendations [15,16,17,18]: (1) lifting loads exceeding 23 kg, (2) prolonged standing for more than two hours, (3) computer- or desk-based tasks, (4) improper postures (e.g., overreaching or working with hands above shoulder level), (5) prolonged sitting in chairs without adequate back support, (6) repetitive work cycles, (7) tasks involving forceful exertion, (8) whole-body vibration, and (9) segmental vibration (e.g., the use of vibrating mechanical tools or equipment). Participants’ cumulative biomechanical risk scores (range: 0–9) were derived from nine individual risk factors and dichotomized into low (<4 factors) and high (≥4 factors) risk.
3. Knowledge, Attitude, and Practice (KAP): The KAP section was developed to evaluate four key aspects, specifically: biomechanical risks associated with lifting, safe manual lifting techniques, the utilization of back support belts, and muscle relaxation and stretching exercises. Knowledge was assessed using 10 “True/False/Don’t know” items. Correct answers were assigned 1 point, while incorrect or don’t know responses got 0 points, resulting in a total score range of 0 to 10. Attitude was evaluated using 10 items on a 4-point rating scale (strongly agree, agree, disagree, and strongly disagree). Scores were assigned from 1 to 4, with reverse scoring applied to negative attitude items. A higher cumulative score, with a maximum of 40, represented a more positive attitude towards safe lifting. Practice consisted of 10 items measured on a 5-point frequency scale (everyday, 3–4 times/week, 1–2 time/week, 1–2 times/month, and never). Scores were assigned from 1 to 5, with reverse scoring applied to negative practice items. The total score was 50, with higher scores representing higher adherence to safe lifting practices. The KAP was divided into three levels based on Bloom’s taxonomy cut-off [19], where a score of 80% or higher was considered high, 60% to 79% was moderate, and below 60% was low.
4. Musculoskeletal Symptoms: The Thai version of the Extended Nordic Musculoskeletal Questionnaire (NMQ-E) [20] was used to evaluate MSDs across five anatomical regions relevant to lifting tasks: the hips/legs, lower back, upper back, neck, and shoulders. For each anatomical region, the questionnaire assessed the presence of abnormal symptoms experienced during the past 12 months. Only participants who reported symptoms within the 12-month period were subsequently asked whether active symptoms were present during the past 7 days. Additionally, the questionnaire evaluated pain severity using a Visual Analog Scale (VAS) pain score ranging from 0 to 10, healthcare and medical care seeking behavior, limitations affecting daily activity and work, and job absence.

The questionnaire was reviewed by three Occupational Medicine Physicians. The Content Validity Index (CVI) was calculated, yielding an Item-level CVI (I-CVI) of 0.977 and a Scale-level CVI (S-CVI) of 0.930, indicating excellent content relevance and representativeness.

### 2.3. Statistical Analysis

Participants reporting musculoskeletal symptoms in any anatomical region within the past 12 months were categorized into the ‘present MSD’ group. Descriptive statistics were presented as frequencies and percentages (N, %), mean ± SD and median and interquartile range (IQR). Categorical variables were compared using the Chi-square test, and the Wilcoxon rank-sum test was utilized for non-parametric continuous data. Item-level comparisons of knowledge and safety practice frequencies were conducted using exploratory univariate Chi-square tests without adjustment for multiple testing.

To further differentiate between overall and recent symptom associations, multinomial logistic regression was employed to compare three distinct groups: no MSDs (reference), 12-month MSDs without 7-day pain, and active 7-day MSDs (12-month symptoms accompanied by 7-day pain). Biomechanical risk was modeled as a continuous variable based on the total number of biomechanical risk factors present. To avoid overfitting and sparse-data bias, a parsimonious model was adopted. The final model retained seven core predictors: age, overtime duration, physical activity, biomechanical risk score, and KAP scores. All statistical analyses were performed using STATA version 16 (StataCorp LLC, College Station, TX, USA). Statistical significance was defined as a *p*-value < 0.05.

### 2.4. Ethical Considerations

This study was approved by the Research Ethics Committee of the Faculty of Medicine, Chiang Mai University, Thailand (study code: COM-2568-0496; research ID: 0496; date of approval: 22 July 2025 to 21 July 2026). Informed consent was obtained for studies involving all volunteers before data collection. Data collection was conducted between 22 July 2025 and 17 March 2026. No participants were enrolled prior to ethical approval on 22 July 2025.

## 3. Results

### 3.1. Participants’ Characteristics and Occupational Characteristics

A total of 99 participants were included in this study. The mean age of the entire sample was 33.4 ± 7.7 years, with a predominant male majority (68.7%). Additionally, most of the participants (79.8%) reported having no underlying diseases. Most participants reported no history of trauma, engaged in physical activity, and participated in hobbies. Regarding occupational characteristics, the median job experience was 7.25 years (P25–P75: 2.5–10.66). The mean overtime work was 3.49 ± 1.8 h. Overall, biomechanical risk exposure was low (<3 risk factors). The most prevalent risk factors were forceful exertion (72.7%), repetitive hand/wrist movements (69.7%), and prolonged standing (61.6%).

Of the 99 participants, 56 (56.6%) reported MSDs. Among present MSD participants, the mean age was 35.1 ± 7.7 years, and most were male. Compared to the no MSD group, present MSD workers were significantly older (*p* = 0.042) and had a higher prevalence of underlying diseases (30.36% vs. 7.0%, *p* = 0.004), which most commonly included hypertension, diabetes and gout. They also reported fewer workdays per week (5.68 ± 0.9 vs. 6.05 ± 0.4 days, *p* = 0.016) and higher overtime duration (3.53 ± 1.5 vs. 3.44 ± 2.2 *p* = 0.022). Furthermore, present MSD workers had significantly higher proportion of biomechanical risks, including improper posture (*p* = 0.001), repetitive hand/wrist movements (*p* = 0.002), forceful exertion (*p* = 0.004), segmental vibration (*p* = 0.018), and computer/desk-based tasks (*p* = 0.037). Conversely, prolonged standing was significantly more prevalent in the no MSD group compared to the present MSD group (83.7% vs. 44.6%, *p* < 0.001). Further details are presented in Table 1 and Table 2.

### 3.2. The Prevalence of MSDs

**Figure 2:**
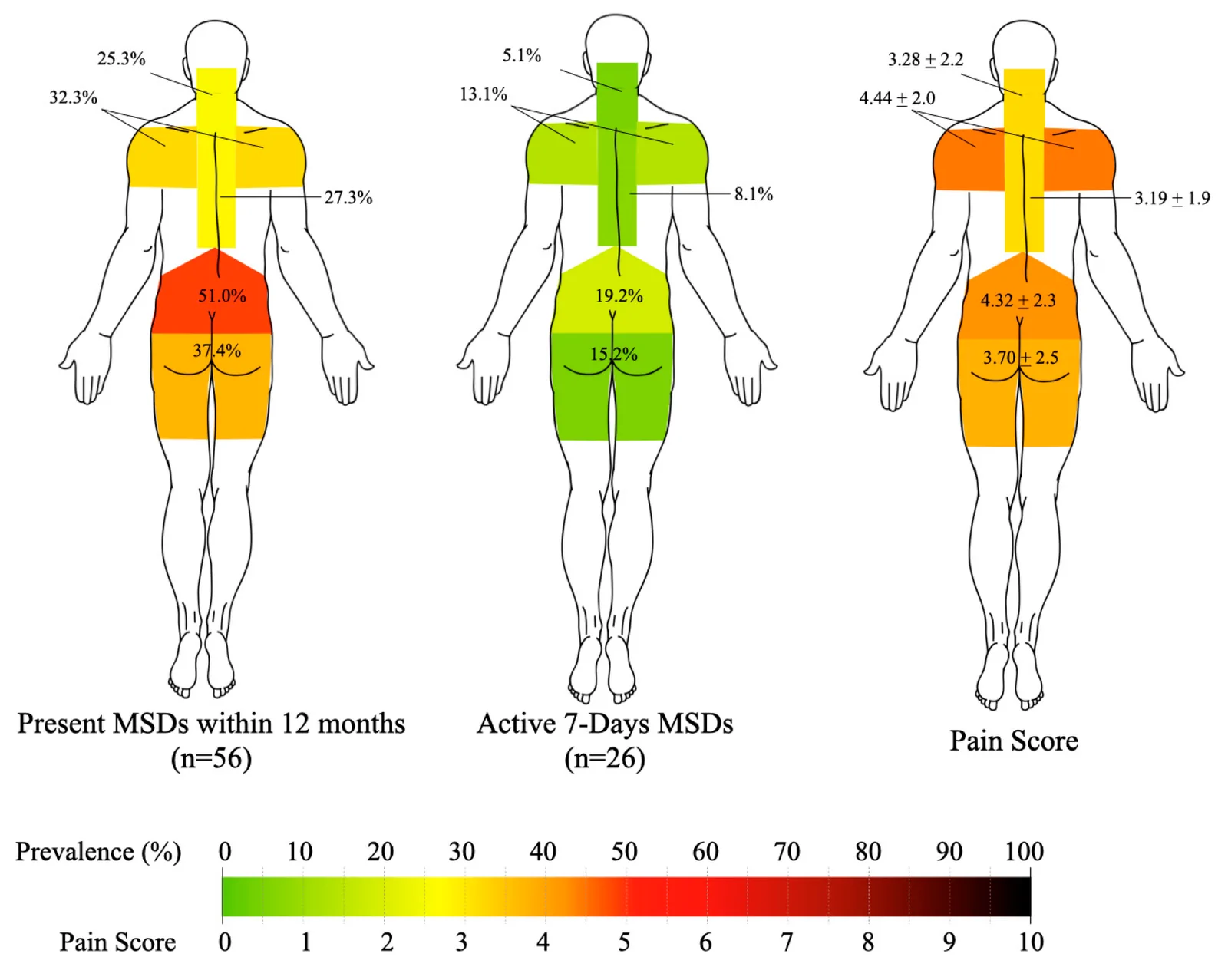
Musculoskeletal symptoms across five regions: 12-month presence of MSDs, active 7-day MSDs, and mean pain scores. The anatomical diagrams illustrate the distribution and intensity of musculoskeletal symptoms across the five body areas: 12-month presence of MSDs (**left**), active 7-day MSDs (**middle**), and mean pain scores with standard deviations (**right**). Prevalence values are shown as percentages (%), and pain scores are presented on a 0–10 scale. The color gradient indicates magnitude, with darker shades representing higher values. The lower back region demonstrated the highest 12-month presence of MSDs (51.0%), followed by the hips/legs (37.4%) and shoulders (32.3%). Active 7-day MSDs were highest in the lower back (19.2%) and hip/leg (15.2%) regions. Mean pain scores were greatest in the shoulder (4.44 ± 2.0) and lower back (4.32 ± 2.3) areas. Original figure created by the authors using Canva Pro (under Canva Content License) and Apple Keynote.

Among the total participants, 56.6% (*n* = 56) experienced MSDs during the last 12 months, and 46.4% of those with 12-month MSDs reported active pain during the past 7 days (26.3% of the total sample, *n* = 26). A total of 62.5% required medication, 39.3% experienced activity limitation, 37.5% reported absence from work, and 26.8% reported job limitation. As shown in Figure 2, the most affected site was the lower back (51%), followed by the hip/leg (37.4%) and shoulder (32.3%). The highest mean pain score was observed in the shoulder (4.44 ± 2.0), followed by the lower back (4.32 ± 2.3) and hip/leg (3.70 ± 2.5).

### 3.3. Knowledge, Attitude and Practice: No MSD Group and Present MSD Group

The overall mean knowledge score was 6.66 ± 1.9 out of a total of 10 points, with the majority of participants categorized as having low knowledge (62.6%). Overall, 78.8% and 47.5% of participants had moderate attitude and practice levels, respectively. The KAP results stratified by MSD status are presented in Table 3. No statistically significant differences between the no MSD and present MSD groups were observed in knowledge or attitude. However, participants in the present MSD group demonstrated significantly poorer lifting practices compared with those in the no MSD group (*p* = 0.025). Additional details are provided in Table 3.

**Figure 3:**
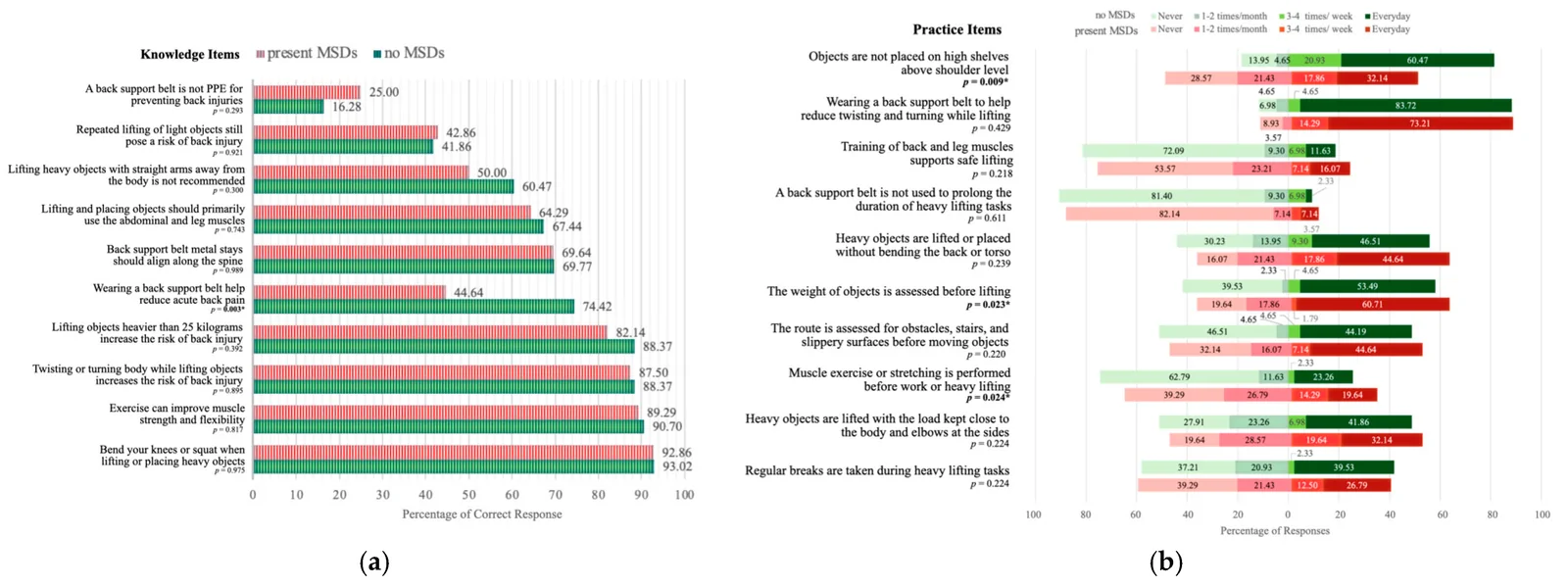
Exploratory comparison of correct responses to the knowledge and Likert scale responses for practice frequency among participants with no MSDs and those with present MSDs. Green indicates participants with no MSDs, and red indicates participants with present MSDs. The asterisk (*) indicates statistical significance at *p* < 0.05. (**a**) Correct responses, showing a significant difference only for the item “Wearing a back support belt helps reduce acute back pain” (*p* = 0.003); all other items are non-significant. (**b**) Frequency of safety practices, showing significant differences between groups in three aspects: objects are not placed on high shelves above shoulder level (*p* = 0.009), muscle exercise or stretching is performed before work or heavy lifting (*p* = 0.024), and assessment of object weight before lifting (*p* = 0.023). All analyses were performed using the Chi-square test.

Item-level analysis revealed a statistically significant difference in the knowledge item regarding whether using a back belt can reduce acute low back pain (*p* = 0.003). In the practice domain, significant differences were identified in lifting objects above shoulder/head level (lifting overhead) (*p* = 0.009), performing muscle strengthening or stretching exercises before work (*p* = 0.024), and assessing object weight before lifting (*p* = 0.023). Detailed item-level comparisons are presented in Figure 3.

### 3.4. Factors Associated with MSDs Among the 12-Month MSD Without 7-Day Pain Group and the Active 7-Day MSD Group

Multinomial logistic regression, using the no MSD group as the reference category, revealed that higher biomechanical risk factor scores were significantly associated with increased likelihood of MSDs during a 12-month MSD without 7-day pain in both the full model and parsimonious model (RRR = 1.60, 95% CI: 1.13 to 2.27, *p* = 0.008) and the parsimonious model (RRR = 1.54, 95% CI: 1.10 to 2.16, *p* = 0.012). Conversely, higher knowledge scores demonstrated a significant protective association against 12-month MSDs without 7-day pain in both full and parsimonious models (RRR = 0.73, 95% CI: 0.54 to 0.98, *p* = 0.039 and RRR = 0.74, 95% CI: 0.56 to 0.99, *p* = 0.045). In the final model, age was significantly associated with MSDs during active 7-day MSDs (RRR = 1.12, 95% CI: 1.02 to 1.23., *p* = 0.014). No other variables included in the model demonstrated statistically significant associations with MSDs. Detailed multinomial regression results are presented in Table 4.

## 4. Discussion

This cross-sectional study investigated the prevalence, biomechanical risk factors, and role of KAP concerning MSDs among manual lifting workers. The findings revealed that 56.6% of the participants experienced MSDs over the past 12 months, with the lower back being the most predominantly affected region. This high prevalence mirrors findings from the Global Burden of Disease 2021 study, which identified LBP as a leading global driver of disability [2]. Furthermore, the study demonstrated a significant positive association between advancing age and the likelihood of reporting acute MSDs within the past 7 days (RRR = 1.12). This is congruent with global disease burden trends, which indicate that the incidence and burden of LBP escalate with age, typically peaking in middle-to-older adulthood [1,21]. Notably, a 30-year global longitudinal study on MSDs indicated that MSD incidence peaks between 35 and 59 years, with a sharp pinnacle at 45 to 49 years, a trend largely dominated by LBP [22].

Regarding occupational risks, the present study identified a robust association between elevated biomechanical risk factor scores and the occurrence of long-term MSDs. However, this association was not statistically significant for active 7-day MSDs. This pattern suggests that biomechanical hazards—such as forceful exertion, improper postures, and heavy lifting—are more strongly correlated with chronic symptoms than with active pain episodes. Notable biomechanical risk factors reported among the workers included forceful exertion, improper postures, and heavy lifting of loads exceeding 23 kg. It is critical to contrast this biomechanical threshold with the current Thai regulatory standards. Under the Ministerial Regulation of Thailand regarding permissible lifting weights, adult male workers (≥18 years) are legally permitted to lift up to 50 kg, while adult female workers are limited to 25 kg [23]. This highlights a significant discrepancy between legally permissible limits and biomechanically safe thresholds. Consequently, workers lifting weights that are entirely within the legal limit (e.g., between 23 kg and 50 kg for adult males) remain exposed to substantial ergonomic hazards. This suggests that relying solely on national legislative limits is insufficient for preventing cumulative musculoskeletal injuries. Systematic reviews and meta-analyses have demonstrated moderate to strong evidence linking mechanical exposures to chronic LBP [5]. With prolonged exposure, these sustained occupational stresses precipitate morphological changes in the intervertebral disc tissue, characterized by reduced water retention and diminished elasticity. Consequently, the discs lose their capacity to effectively absorb and distribute pressure, which increases the risk of degenerative tears in the annulus fibrosus and subsequent displacement or leakage of the nucleus pulposus (disc herniation) [24]. This pathophysiological mechanism is strongly supported by recent meta-analyses, which report significantly elevated risks of chronic LBP associated with combined mechanical exposures (OR = 2.2; 95% CI 1.4–3.6), lifting or carrying loads (OR = 1.7; 95% CI 1.4–2.2), and working in non-neutral postures (OR = 1.5; 95% CI 1.2–1.9) [5]. Moreover, the significant difference in improper postures between the present MSD and no MSD groups (*p* = 0.001) further underscores the impact of non-neutral alignments on spinal health [25].

The assessment of KAP provided critical insights into worker behaviors and their impact on musculoskeletal health. While unadjusted bivariate analyses showed no significant differences in KAP scores between the present MSD and no MSD groups, baseline knowledge regarding safe lifting was suboptimal. However, after adjusting for potential confounders and accounting for acute, short-term cases in the multivariate multinomial logistic regression model, an inverse association between knowledge scores and 12-month MSK symptoms emerged. This suggests that safety knowledge acts as a long-term preventive mechanism, underscoring the need for comprehensive worker education. However, the insufficiency of knowledge alone to mitigate MSK risks is well-documented; previous studies indicate that education or ergonomic adjustments independently do not effectively prevent LBP [7,26]. Instead, multifaceted interventions combining education with active exercise programs have proven to be the most efficacious strategy for LBP prevention [7,8]. The lack of pre-work muscle stretching and strengthening among MSD workers is particularly concerning, as diminished back extensor endurance is a pronounced deficit in manual workers with chronic LBP [11]. Consistently, our study also found that MSD participants exhibited significantly poorer adherence to safe lifting practices in three key hazardous behaviors: lifting objects above shoulder level, failing to assess object weight prior to lifting, and neglecting pre-work muscle strengthening or stretching exercises. These findings dictate that a multidimensional, integrated intervention strategy, encompassing both behavioral education and physical conditioning, must be implemented to effectively safeguard worker health.

Additionally, the study showed misconceptions regarding the efficacy of back support belts in reducing acute LBP. Current systematic reviews and clinical guidelines underscore a lack of conclusive evidence supporting the use of back belts for the primary prevention of occupational LBP or the reduction in associated sick leave [8,9]. Relying on passive supports such as back belts may paradoxically induce a false sense of security, potentially encouraging workers to engage in risk-taking behaviors, such as lifting loads that exceed their physical capacity while neglecting proper biomechanics [8,9].

Consequently, comprehensive workplace health and safety programs must transcend passive interventions and basic educational training. Strategic engineering and administrative controls must be enforced to mitigate hazardous biomechanical exposures; specifically, ergonomics programs should regulate lifting weights, task frequency, and postural demands while integrating mechanical assistive devices to minimize physical strain [27,28,29]. Furthermore, age-related vulnerabilities should be addressed by modifying physically demanding tasks for older employees. Continuous safe lifting training must be integrated with behavioral interventions by implementing active, exercise-based interventions to foster muscle endurance and functional resilience. This combined strategy fosters long-term knowledge, improves worker working conditions, and translates safety theory into safe daily practices, thereby sustainably reducing the occupational burden of MSDs [8,11].

These findings highlight key public health and occupational safety implications for industrial workforce protection. To reduce WMSDs, strategies must shift from passive safety training to practice-based instruction. Policy makers and practitioners should enforce age-tailored ergonomic regulations with biomechanical controls and establish surveillance systems that separate acute from chronic injury patterns. These multi-level measures are essential to safeguard worker health.

This research’s strength is in its goal to comprehend problems and develop sustainable solutions to ergonomic challenges faced by workers. We examined the prevalence of diseases and pain sites, the time of disease onset in both acute and chronic stages, and the associated risk factors in each phase, enabling us to pinpoint specific intervention opportunities. We delivered systematic feedback to integrate the knowledge and attitudes of workers into practice, thereby advancing occupational health within enterprises in Thailand, where legislation has not yet explicitly addressed the management of ergonomic risk factors.

This study has certain limitations that should be acknowledged. First, owing to methodological constraints, the disease data were acquired through retrospective inquiries, potentially introducing recall bias. Furthermore, the study’s relatively small sample size and reliance on convenience sampling from specific manufacturing facilities limit overall sample representativeness. Consequently, the findings should be interpreted with caution when generalizing the conclusions to broader industrial populations. When applying this dataset in different contexts, it is essential to account for variations in demographic data. A further drawback of this research is that, owing to operational constraints with external agencies in data analysis, we have only acquired the preliminary results of the causative factor analysis and have not yet pursued the long-term application of the study findings. Planning to analyze and monitor the application of research findings, particularly through action research methodologies for further development, would be advantageous for validating hypotheses and attaining more definitive problem-solving results.

## 5. Conclusions

This study underscores a substantial occupational burden of MSDs, predominantly LBP, among heavy manual lifting workers. This high prevalence is primarily driven by cumulative biomechanical hazards. Furthermore, older age significantly escalates acute 7-day risks. While higher knowledge demonstrates a significant protective association against long-term MSDs, a critical knowledge-to-practice gap remains evident in workers’ suboptimal execution of safe lifting practices. Consequently, traditional workplace safety strategies that rely solely on passive educational training are inadequate. Strategic emphasis must be redirected toward integrated ergonomics programs that combine targeted biomechanical risk reductions specifically tailored to accommodate aging workers with active, practice-based educational training to foster behavioral modification and functional resilience among high-risk workers.

## Figures and Tables

**Table 1:** Demographic and occupational characteristics of participants overall and compared between the no MSD and present MSD groups.

Characteristic	Total *N* = 99	MSDs		*p*
		No MSDs (*n* = 43)	Present MSDs (*n* = 56)	
Age (years)				**0.042 ^a^**
Mean ± SD	33.4 ± 7.7	31.9 ± 7.3	35.1 ± 7.7	
Median [P25–P75]	32 [27.5–36.5]	30 [25–35]	32.5 [27.25–37.75]	
Height (cm)				0.358 ^a^
Mean ± SD	165.7 ± 8.5	166.6 ± 7.7	165 ± 9.0	
Median [P25–P75]	167 [158–172]	168 [160–172]	165 [158–172.5]	
Weight (kg)				0.846 ^b^
Mean ± SD	70.3 ± 15.8	69.9 ± 15.9	70.6 ± 15.8	
Median [P25–P75]	69 [58–80]	70 [58–80.9]	69 [60–76.5]	
BMI (kg/m^2^)				0.619 ^a^
Mean ± SD	25.7 ± 5.3	25.0 ± 4.7	26.3 ± 5.7	
Median [P25–P75]	24.44 [22.57–29.05]	24.22 [22.23–29.07]	24.71 [22.59–28.68]	
Sex, n (%)				0.522 ^c^
Male	68 (68.7)	31 (72.1)	37 (66.1)	
Female	31 (31.3)	12 (27.9)	19 (33.9)	
Education, *n* (%)				0.533 ^c^
Primary school	1 (1.0)	0 (0.0)	1 (1.8)	
Secondary school	65 (65.7)	27 (62.8)	38 (67.9)	
Bachelor’s degree or higher	33 (33.3)	16 (37.2)	17 (30.3)	
Underlying disease(s), *n* (%)				**0.004 ^c^**
No	79 (79.8)	40 (93.0)	39 (69.6)	
Yes ^d^	20 (20.2)	3 (7.0)	17 (30.4)	
Smoking habits, *n* (%)				0.898 ^c^
Non-smoker	60 (60.6)	27 (62.8)	33 (58.9)	
Ex-smoker	21 (21.2)	9 (20.9)	12 (21.4)	
Active smoker	18 (18.2)	7 (16.3)	11 (19.7)	
Physical activities, *n* (%)				0.383 ^c^
No	48 (48.5)	23 (53.5)	25 (44.6)	
Yes	51 (51.5)	20 (46.5)	31 (55.4)	
Hobbies, *n* (%)				0.756 ^c^
No	42 (42.4)	19 (44.2)	23 (41.1)	
Yes ^e^	57 (57.6)	24 (55.8)	33 (58.9)	
Job experience (years)				0.463 ^c^
Mean ± SD	7.95 ± 6.5	7.41 ± 6.7	8.36 ± 6.3	
Median [P25–P75]	7.25 [2.5–10.66]	7.33 [1.25–10.66]	7.13 [4.21–10.33]	
Workdays per week				**0.016 ^a^**
Mean ± SD	5.84 ± 0.8	6.05 ± 0.4	5.68 ± 0.9	
Median [P25–P75]	6 [5–6]	6 [6–6]	6 [5–6]	
Working hours per day				0.678 ^a^
Mean ± SD	8.11 ± 1.3	7.91 ± 1.4	8.26 ± 1.2	
Median [P25–P75]	8 [8–8]	8 [8–8]	8 [8–8]	
Overtime work				
No	13 (13.1)	8 (18.6)	5(8.9)	0.158 ^c^
Yes	86 (86.9)	35 (81.4)	51 (91.1)	
Overtime duration (hours)				**0.022 ^a^**
Mean ± SD	3.49 ± 1.8	3.44 ± 2.2	3.53 ± 1.5	
Median [P25–P75]	3 [2.4–4]	3 [2.4–3]	3 [2.67–4]	
Secondary employment, *n* (%)				0.411 ^c^
No	92 (92.9)	41 (95.4)	51 (91.1)	
Yes	7 (7.1)	2 (4.6)	5 (8.9)	

Note: Data are expressed as the mean ± SD, median [P25–P75], or *n* (%). ^a^ Analyzed using the Wilcoxon rank; ^b^ analyzed using the unpaired *t*-test; ^c^ analyzed using the Chi-square. ^d^ Underlying included hypertension, diabetes, dyslipidemia, allergic rhinitis, herniated nucleus pulposus and gout. ^e^ Hobbies included gardening, sports, cycling, playing musical instruments, handicrafts/needlework, and gaming/prolonged computer use. Abbreviation: BMI: body mass index; SD: standard deviation; MSDs: musculoskeletal disorders.

**Table 2:** Biomechanical risk factors and back belt use of participants overall and compared between the no MSD and present MSD groups.

Characteristic	Total *N* = 99	MSDs		*p*
		No MSDs (*n* = 43)	Present MSDs (*n* = 56)	
Individual biomechanical risk factors, *n* (%)				
Lifting (>23 kg.)	31 (31.31)	9 (20.9)	22 (39.3)	0.051 ^a^
Prolonged standing	61 (61.6)	36 (83.7)	25 (44.6)	**<0.001 ^a^**
Computer/desk-based tasks	44 (44.4)	14 (32.6)	30 (53.6)	**0.037 ^a^**
Improper posture	53 (53.5)	15 (34.9)	38 (67.9)	**0.001 ^a^**
Prolonged sitting	14 (14.1)	5 (11.6)	9 (16.1)	0.529 ^a^
Repetitive hand/wrist movement	69 (69.7)	23 (53.5)	46 (82.1)	**0.002** ^a^
Forceful exertion	72 (72.7)	25 (58.1)	47 (83.9)	**0.004** ^a^
Whole-body vibration	25 (25.2)	9 (20.9)	16 (28.6)	0.386 ^a^
Segmental vibration	20 (20.2)	4 (9.3)	16 (28.6)	**0.018** ^a^
Total biomechanical risk factor score				
Mean ± SD	3.93 ± 1.83	3.26 ± 1.80	4.45 ± 1.68	**0.001 ^b^**
Median [P25–P75]	4 [3–5]	3 [2–5]	4 [3.5–6] [21]	
Biomechanical risk category *, *n* (%)				
Low	63 (63.6)	32 (74.4)	31 (55.4)	0.051 ^a^
High	36 (36.4)	11 (25.6)	25 (44.6)	
Back belt use, *n* (%)				0.112 ^a^
No	86 (86.9)	40 (93.0)	46 (82.1)	
Yes	13 (13.1)	3 (7.0)	10 (17.9)	

Note: Data are expressed as the mean ± SD, median [P25–P75], or *n* (%). * Biomechanical risk categorized using a cut-off of ≥4 risk factors for high risk. ^a^ Analyzed using the Chi-square; ^b^ analyzed using the unpaired *t*-test. Abbreviation: MSDs: musculoskeletal disorders.

**Table 3:** Comparison of knowledge, attitude, and practice scores and levels between the no MSD and present MSD groups.

	Knowledge			Attitude			Practice		
	No MSDs	Present MSDs	*p*	No MSDs	Present MSDs	*p*	No MSDs	Present MSDs	*p*
Mean ± SD	6.91 ± 2.1	6.48 ± 1.8	0.080 ^a^	29.2 ± 2.8	29.6 ± 2.5	0.342 ^a^	26.8 ± 7.2	28.8 ± 6.4	0.197 ^a^
Median [P25–P75]	7 [6–8]	7 [6–7]		29 [27–31]	29.5 [28–31]		27 [21–33]	27 [23–34.5]	
Group, *n* (%)			0.233 ^b^			0.925 ^b^			**0.025 ^b^**
Low	31 (72.1)	31 (55.3)		0 (0.0)	0 (0.0)		7 (16.3)	11 (19.6)	
Moderate	11 (25.6)	23 (41.1)		34 (79.1)	44 (78.6)		15 (34.9)	32 (57.1)	
High	1 (2.3)	2 (3.6)		9 (20.9)	12 (21.4)		21 (48.8)	13 (23.2)	

^a^ Analyzed using the Wilcoxon rank; ^b^ analyzed using the Chi-square.

**Table 4:** Multinomial logistic regression analysis of factors associated with the 12-month MSD without 7-day pain group and the active 7-day MSD group.

Factors	Model 1: Full Model			Model 2: Parsimonious Model /Final Model		
	RRR	[95% CI]	*p*	RRR	[95% CI]	*p*
12-month MSDs without 7-day pain						
Age (years)	1.06	[0.96, 1.17]	0.250	1.08	[0.98, 1.19]	0.129
Overtime duration (hours)	0.96	[0.69, 1.34]	0.809	0.96	[0.68, 1.34]	0.799
Biomechanical risk factors **	1.60	[1.13, 2.27]	0.008 *	1.54	[1.10, 2.16]	0.012 *
Knowledge total score	0.73	[0.54, 0.98]	0.039 *	0.74	[0.56, 0.99]	0.045 *
Attitude total score	1.11	[0.89, 1.39]	0.355	1.12	[0.90, 1.40]	0.308
Practice total score	1.09	[0.98, 1.20]	0.102	1.07	[0.98, 1.17]	0.128
Physical activities (Ref: No)	1.08	[0.31, 3.81]	0.905	0.96	[0.28, 3.27]	0.943
Underlying disease (Ref: No)	2.68	[0.37, 19.38]	0.330	–		
Sex (Ref: Male)	2.37	[0.55, 10.15]	0.244	–		
Active 7-day MSDs						
Age (years)	1.08	[0.98, 1.19]	0.115	1.12	[1.02, 1.23]	0.014 *
Overtime duration (hours)	0.98	[0.68, 1.40]	0.900	0.99	[0.70, 1.39]	0.951
Biomechanical risk factors **	1.36	[0.95, 1.96]	0.097	1.28	[0.91, 1.79]	0.152
Knowledge total score	1.05	[0.73, 1.51]	0.790	1.02	[0.73, 1.42]	0.925
Attitude total score	1.08	[0.85, 1.36]	0.529	1.09	[0.88, 1.36]	0.413
Practice total score	1.00	[0.90, 1.11]	0.985	1.00	[0.91, 1.10]	0.964
Physical activities (Ref: No)	1.93	[0.53, 7.07]	0.321	1.51	[0.46, 4.99]	0.496
Underlying disease (Ref: No)	9.35	[1.50, 58.16]	0.017 *	–		
Sex (Ref: Male)	2.73	[0.68, 10.88]	0.155	–		

* p < 0.05. ** Biomechanical risk was modeled as a continuous score.

## Data Availability

The data presented in this study are available on request from the corresponding author due to ethical considerations and the requirement for approval from the relevant authorities before data sharing.

## Supplementary Materials

The following supporting information can be downloaded at: https://www.mdpi.com/article/10.3390/ijerph23081041/s1, File S1: STROBE Statement—checklist of items that should be included in reports of observational studies.

## Abbreviations

The following abbreviations are used in this manuscript:

BMI	Body mass index
KAP	Knowledge, attitude, and practice
LBP	Low back pain
MSDs	Musculoskeletal disorders
WMSDs	Work-related musculoskeletal disorders
