# Supplementary material for: The Knowledge-to-Practice Gap in Safe Lifting and Biomechanical Risks as Determinants of Musculoskeletal Disorders in Manual Material Handling
Source: Int J Environ Res Public Health. 2026 Aug 11;23(8):1041. doi: 10.3390/ijerph23081041 (PMC13513384; doi:10.3390/ijerph23081041)
Supplement: Supplementary file 1 [file ijerph-23-01041-s001.zip › ijerph-4423862-supplementary File S1.pdf]

STROBE Statement—checklist of items that should be included in reports of observational studies

	Item No.	Recommendation	Page No.	Relevant text from manuscript
**Title and abstract**	1	(*a*) Indicate the study's design with a commonly used term in the title or the abstract	1	"A cross-sectional study surveyed 99 industrial manual lifting workers within the manufacturing factories..."
		(*b*) Provide in the abstract an informative and balanced summary of what was done and what was found	1	"This study investigated the complex interfaces between knowledge, attitude and practice (KAP), biomechanical risks, and musculoskeletal disorders (MSDs)..." “The 12-month prevalence of MSDs was 56.6%, predominantly affecting the lower back (51.0%)….”
**Introduction**				
Background/rationale	2	Explain the scientific background and rationale for the investigation being reported	2-3	"Work-related musculoskeletal disorders (WMSDs) represent a preeminent global public health challenge... a comprehensive understanding of KAP regarding manual lifting and back belt use, alongside their interrelationships... is imperative for designing sustainable, practical interventions."
Objectives	3	State specific objectives, including any prespecified hypotheses	3	"Therefore, this cross-sectional study primarily aimed to investigate the relationships of KAP regarding manual lifting and back support belt use and the occurrence of musculoskeletal disorder (MSDs) among heavy manual handling workers."
**Methods**				
Study design	4	Present key elements of study design early in the paper	3	"2.1 Study design and Participants: A cross-sectional study was conducted among industrial workers from manufacturing factories..."
Setting	5	Describe the setting, locations, and relevant dates, including periods of recruitment, exposure, follow-up, and data collection	3	"...from manufacturing factories in Chiang Mai and Lamphun provinces... The study period ranged from 22 July 2025 to 17 March 2026."
Participants	6	(*a*) *Cohort study*—Give the eligibility criteria, and the sources and methods of selection of participants. Describe methods of follow-up *Case-control study*—Give the eligibility criteria, and the sources and methods of case ascertainment and control	3	"Inclusion criteria were individuals aged 20 years old or older, working in Chiang Mai or Lamphun provinces, understanding in Thai, and being willing to join study... A convenience sampling strategy was employed via open invitations distributed by the occupational health and safety units and human resources departments within each workplace..."

		selection. Give the rationale for the choice of cases and controls<br>*Cross-sectional study*—Give the eligibility criteria, and the sources and methods of selection of participants		
		(*b*) *Cohort study*—For matched studies, give matching criteria and number of exposed and unexposed<br>*Case-control study*—For matched studies, give matching criteria and the number of controls per case	N/A	Not applicable (Not a matched study).
Variables	7	Clearly define all outcomes, exposures, predictors, potential confounders, and effect modifiers. Give diagnostic criteria, if applicable	4-5	Variables defined in Section 2.2: 1. Demographic data, 2. Working conditions (Biomechanical risk factors across 9 domains), 3. KAP, 4. Musculoskeletal symptoms (present MSDs).
Data sources/ measurement	8*	For each variable of interest, give sources of data and details of methods of assessment (measurement). Describe comparability of assessment methods if there is more than one group	4-5	"The Thai version of the Extended Nordic Musculoskeletal Questionnaire (NMQ-E)<br>was used to evaluate MSDs... KAP section was developed to evaluate four key aspects... The Content Validity Index (CVI) was calculated..."
Bias	9	Describe any efforts to address potential sources of bias	12	(Discussed in Limitations) "...owing to methodological constraints, the disease data were acquired through retrospective inquiries, potentially introducing recall bias."
Study size	10	Explain how the study size was arrived at	3	"Sample size was estimated using one-sample proportion test. The target prevalence (P1 = 65.6%) was derived from a recent global meta-analysis... with a two-sided α=0.05, and 80% power, the minimum sample size was 79 participants. To account for a potential 25% dropout or incomplete response rate, the target sample size was established at a minimum of 99 participants."

Continued on next page

Quantitative variables	11	Explain how quantitative variables were handled in the analyses. If applicable, describe which groupings were chosen and why	4-5	"Participants' cumulative biomechanical risk scores (range: 0–9) were derived from nine individual risk factors and dichotomized into low (<4 factors) and high (≥4 factors) risk." & "The level of KAP was divide into three levels based on Bloom's taxonomy cut-off... 80% or higher was considered high..."
Statistical methods	12	(*a*) Describe all statistical methods, including those used to control for confounding	5	"Descriptive statistics were presented as frequencies and percentages... Categorical variables were compared using the Chi-square test, and the Wilcoxon rank-sum test... Multinomial Logistic Regression was employed to compare three distinct groups..."
		(*b*) Describe any methods used to examine subgroups and interactions	5	"Multinomial Logistic Regression was employed to compare three distinct groups: No MSDs (reference), 12-month MSDs without 7-day pain, and active 7-day MSDs (12-month symptoms accompanied by 7-day pain)."
		(*c*) Explain how missing data were addressed	3	"Participants with incomplete data were excluded."
		(*d*) *Cohort study*—If applicable, explain how loss to follow-up was addressed<br>*Case-control study*—If applicable, explain how matching of cases and controls was addressed<br>*Cross-sectional study*—If applicable, describe analytical methods taking account of sampling strategy	N/A	Not applicable (Convenience sampling without survey weights was used).
		(*e*) Describe any sensitivity analyses	N/A	Not applicable (No sensitivity analyses were performed).
**Results**				
Participants	13*	(a) Report numbers of individuals at each stage of study—eg numbers potentially eligible, examined for eligibility, confirmed eligible, included in the study, completing follow-up, and analysed	4	"A total of 102 workers submitted questionnaires; following the exclusion of 3 incomplete submissions, a final sample of 99 valid responses (N =99) was analyzed."
		(b) Give reasons for non-participation at each stage	3	"A total of 102 workers submitted questionnaires; following the exclusion of 3 incomplete submissions, a final sample of 99 valid responses (N =99) was analyzed."
		(c) Consider use of a flow diagram	4	Figure 1
Descriptive data	14*	(a) Give characteristics of study participants (eg demographic, clinical, social) and information on exposures and potential confounders		

		(b) Indicate number of participants with missing data for each variable of interest	3	"...following the exclusion of 3 incomplete submissions, a final sample of 99 valid responses (N =99) was analyzed."
		(c) *Cohort study*—Summarise follow-up time (eg, average and total amount)	N/A	Not applicable (Cross-sectional design).
Outcome data	15*	*Cohort study*—Report numbers of outcome events or summary measures over time		
		Case-control study—Report numbers in each exposure category, or summary measures of exposure		
		Cross-sectional study—Report numbers of outcome events or summary measures	8	"Among the total participant, 56.6% (n=56) experienced in with MSDs during the past 12 months, and 46.4% of those with 12-month MSDs reported active pain during the past 7 days (26.3% of the total sample, n=26)."
Main results	16	(*a*) Give unadjusted estimates and, if applicable, confounder-adjusted estimates and their precision (eg, 95% confidence interval). Make clear which confounders were adjusted for and why they were included	10	"...higher biomechanical risk factor scores were significantly associated with increased likelihood of MSDs during the 12-month MSDs without 7-day pain... in the parsimonious model (RRR=1.54, 95% CI: 1.10 to 2.16, p=0.012)."
		(*b*) Report category boundaries when continuous variables were categorized	4-5	"Participants' cumulative biomechanical risk scores (range: 0–9) were derived from nine individual risk factors and dichotomized into low (<4 factors) and high (≥4 factors) risk." & "The level of KAP was divide into three levels based on Bloom's taxonomy cut-off... 80% or higher was considered high..."
		(*c*) If relevant, consider translating estimates of relative risk into absolute risk for a meaningful time period	N/A	Not applicable

Continued on next page

Other analyses	17	Report other analyses done—eg analyses of subgroups and interactions, and sensitivity analyses	9	"Item-level analysis revealed a statistically significant difference in the knowledge item regarding whether using back belt can reduce acute low back pain (p = 0.003)..." (Illustrated in Figure 3)
**Discussion**				
Key results	18	Summarise key results with reference to study objectives	10-11	"The findings revealed that 56.6% of the participants experienced MSDs over the past 12 months... Furthermore, the study demonstrated a significant positive association between advancing age and the likelihood of reporting acute MSDs..."
Limitations	19	Discuss limitations of the study, taking into account sources of potential bias or imprecision. Discuss both direction and magnitude of any potential bias	12	"This study has certain limitations that should be acknowledged. …"
Interpretation	20	Give a cautious overall interpretation of results considering objectives, limitations, multiplicity of analyses, results from similar studies, and other relevant evidence	12	"This temporal divergence suggests that biomechanical hazards... primarily exert a cumulative, chronic impact rather than acting as immediate triggers... This suggests safety knowledge acts as a long-term preventive mechanism..."
Generalisability	21	Discuss the generalisability (external validity) of the study results	12	"...reliance on convenience sampling from specific manufacturing facilities limit overall sample representativeness. Consequently, the findings should be interpreted with caution when generalizing the conclusions to broader industrial populations."
**Other information**				
Funding	22	Give the source of funding and the role of the funders for the present study and, if applicable, for the original study on which the present article is based	13	"Funding: This research received no external funding"

*Give information separately for cases and controls in case-control studies and, if applicable, for exposed and unexposed groups in cohort and cross-sectional studies.

Note: An Explanation and Elaboration article discusses each checklist item and gives methodological background and published examples of transparent reporting. The STROBE checklist is best used in conjunction with this article (freely available on the Web sites of PLoS Medicine at http://www.plosmedicine.org/, Annals of Internal Medicine at http://www.annals.org/, and Epidemiology at http://www.epidem.com/). Information on the STROBE Initiative is available at www.strobe-statement.org.
